# Influence of pharmacokinetics‐related gene polymorphisms on plasma levels of clozapine and its metabolites in Japanese patients with schizophrenia

**DOI:** 10.1002/pcn5.70164

**Published:** 2025-07-30

**Authors:** Shinya Kinoshita, Hideyuki Motohashi, Keiichiro Nishida, Seiichiro Tarutani, Tetsufumi Kanazawa, Junya Nagai

**Affiliations:** ^1^ Department of Neuropsychiatry, Faculty of Medicine Osaka Medical and Pharmaceutical University Takatsuki Osaka Japan; ^2^ Department of Pharmaceutics, Faculty of Pharmacy Osaka Medical and Pharmaceutical University Takatsuki Osaka Japan; ^3^ Department of Psychiatry, Shin‐Abuyama Hospital Osaka Institute of Clinical Psychiatry Takatsuki Osaka Japan

**Keywords:** clozapine, drug‐metabolizing enzymes, drug transporters, polymorphism, schizophrenia

## Abstract

**Aim:**

Clozapine is an atypical antipsychotic drug that is most effective against treatment‐resistant schizophrenia and causes serious adverse effects, including agranulocytosis. We examined the relationships between age, sex, genetic polymorphisms, and the plasma concentrations of clozapine and its metabolites (N‐desmethylclozapine and clozapine N‐oxide) in Japanese patients with schizophrenia.

**Methods:**

DNA was isolated from the peripheral blood samples of 27 patients with schizophrenia receiving clozapine maintenance treatment, and the pharmacokinetics‐related genes (*CYP1A2*, *CYP2B6*, *CYP2C19*, *CYP2D6*, *CYP3A5*, *ABCG2*, and *SLCO1B1*) were genotyped. The plasma concentrations of clozapine, N‐desmethylclozapine, and clozapine N‐oxide were measured using liquid chromatography–tandem mass spectrometry.

**Results:**

The plasma concentrations of clozapine and its two major metabolites, N‐desmethylclozapine and clozapine‐N‐oxide, showed significant positive correlations with the daily dose. Neither age nor sex substantially influenced the dose‐adjusted plasma concentrations of clozapine or its two metabolites. The plasma concentrations of clozapine and its two metabolites did not notably differ among the genotypes of cytochrome P450 (CYP): *1A2*, *CYP2B6*, *CYP2C19*, *CYP2D6*, *CYP3A5*, and *ABCG2*. In contrast, the dose‐adjusted plasma concentration of N‐desmethylclozapine was significantly higher in patients with the *SLCO1B1* 521C allele than in those with the 521T/T genotype; the concentrations of clozapine and clozapine N‐oxide were not affected by the genetic polymorphisms.

**Conclusion:**

The *SLCO1B1* T521C polymorphism may strongly impact the pharmacokinetics of N‐desmethylclozapine, a major metabolite of clozapine. Therefore, the *SLCO1B1* polymorphisms and plasma levels of N‐desmethylclozapine as well as clozapine in patients with schizophrenia should be assayed for optimizing clozapine therapy.

## INTRODUCTION

Schizophrenia is a chronic psychiatric disorder, characterized by symptoms such as delusions, hallucinations, and cognitive impairments, affecting approximately 1% of the global population. Approximately 30% of patients with schizophrenia have treatment‐resistant schizophrenia (TRS), which poses a major therapeutic challenge in psychiatry.^1^, ^2^ Clozapine was approved by the US Food and Drug Administration (FDA) in 1989 and is the only FDA‐approved drug for treatment‐resistant schizophrenia.^3^ Clozapine reduces psychotic behaviors in patients with schizophrenia and schizoaffective disorder. The medication has been widely prescribed in accordance with the appropriate local guidelines that support safe and effective use.^4^, ^5^, ^6^ Biomarkers for treatment with antipsychotics, including clozapine, have been studied for use in differential diagnosis, predicting drug response, prognosis, as well as monitoring treatment and side‐effects.

Clozapine has several potential side‐effects, including severe neutropenia or agranulocytosis, seizures, severe gastrointestinal hypomobility, myocarditis, pneumonia, and hypotension. The etiology of the side‐effects of clozapine is currently unknown; however, genetic factors may contribute to these adverse reactions. A genome‐wide association study (GWAS) identified the roles of HLA‐DQB1 and HLA‐B in clozapine‐induced agranulocytosis/granulocytopenia.^7^ Another GWAS subsequently identified an association between clozapine‐induced agranulocytosis/granulocytopenia and rs149104283, which is located within a genomic region covering *SLCO1B3*, *SLCO1B7*, and *SLCO1B1*, members of a family of hepatic transporter genes.^8^

Clozapine is metabolized, mainly in the liver, into two major metabolites: the pharmacologically active N‐desmethylclozapine and the inactive clozapine N‐oxide. Various cytochrome P450 (CYP) enzymes, including CYP1A2, CYP2C19, CYP2D6, and CYP3A4, are involved in the metabolism of clozapine.^9^, ^10^, ^11^, ^12^, ^13^ Polymorphic CYP enzymes may be responsible for the considerable variation in the plasma levels of clozapine among patients administered the same dose.

Patients initiating clozapine therapy must have a baseline absolute neutrophil count and should be regularly monitored. The recommended therapeutic plasma clozapine concentration is 350–600 ng/mL.^14^ The optimal clozapine dosage is determined through monitoring the therapeutic response, adverse effects, and the absolute neutrophil count. The results of genetic tests before clozapine treatment provide the information on the pharmacokinetic and pharmacodynamic status of the patient that is necessary for optimizing the clozapine dosage to achieve appropriate therapeutic outcomes and minimize adverse effects.

We investigated the effects of age, sex, and genetic polymorphisms in CYP isoforms (*CYP1A2*, *CYP2B6*, *CYP2C19*, *CYP2D6*, and *CYP3A5*) and drug transporters (*ABCG2* and *SLCO1B1*) on the plasma concentrations of clozapine, N‐desmethylclozapine, and clozapine N‐oxide in Japanese patients with schizophrenia. Clozapine is a hepatically metabolized drug, and considering the balance between its absorption and excretion, we selected ABCG2, the key gene responsible for drug excretion, and SLCO1B1, the key gene responsible for hepatic uptake. We hypothesized that this selection would enable the investigation of the functional interplay between these two genes in regulating drug concentrations.

## METHODS

### Patients

We enrolled 27 patients (14 women and 13 men) more than 1month before the start of clozapine treatment. The patients received oral clozapine as maintenance treatment. The characteristics of the patients are listed in Table 1.

**Table 1:** Demographic and clinical characteristics of patients.

Number of patients	27	
Sex		
Male	13	
Female	14	
Age (years)	42.4 ± 9.5	(26–60)
Clozapine dose (mg/day)	354 ± 167	(75–600)
Bodyweight (kg)	66.6 ± 9.5	(42.1–86.9)
Smokers	5	
Plasma level (ng/mL)		
Clozapine	733 ± 467	(122–1956)
N‐desmethylclozapine	311 ± 234	(56–1069)
Clozapine N‐oxide	86 ± 44	(18–195)
Genotype group		
*CYP1A2* C‐163A	*1/*1 : *1/*1F : *1F/*1F = 3 : 12 : 12	
*CYP2B6* G516T	*1/*1 : *1/*9 : *9/*9 = 20 : 6 : 1	
*CYP2B6* A785G	*1/*1 : *1/*4 : *4/*4 = 13 : 12 : 2	
*CYP2C19* G636A	*1/*1 : *1/*3 = 21 : 6	
*CYP2C19* G681A	*1/*1 : *1/*2 : *2/*2 = 15 : 10 : 2	
*CYP2D6* T733C	*1/*1 : *1/*2 : *2/*2 = 1 : 9 : 17	
*CYP2D6* C100T	*1/*1 : *1/*10 : *10/*10 = 12 : 14 : 1	
*CYP3A5* A6986G	*1/*3 : *3/*3 = 10 : 17	
*ABCG2* C421A	*1/*1 : *1/*2 : *2/*2 = 10 : 15 : 2	
*SLCO1B1* A388G	*1/*1 : *1/*37 : *37/*37 = 3 : 11 : 13	
*SLCO1B1* T521C	*1/*1 : *1/*5 = 22 : 5	

*Note*: Data are reported as the number or mean ± standard deviation (range).

### Measurement of clozapine and its metabolites

Clozapine is metabolized in the liver to clozapine, N‐desmethylclozapine, and clozapine N‐oxide.^15^, ^16^ The concentrations of these three compounds in the blood were measured following previously reported methods^17^ with some modifications. Clozapine and N‐desmethylclozapine were purchased from Tokyo Chemical Industry Co. Ltd. (Tokyo, Japan). Clozapine N‐oxide was purchased from Sigma‐Aldrich (St. Louis, MO, USA). Clozapine‐d_8_ was purchased from Santa Cruz Biotechnology (Dallas, TX, USA) and used as an internal standard (IS) for measuring the concentrations of clozapine and its metabolites. An LCMS‐8045 triple–quadrupole tandem mass spectrometer (Shimadzu Co., Kyoto, Japan) and a Nexera ultra‐high‐performance liquid chromatography system (Shimadzu Co.) were used as the LC–MS/MS system. A SunShell RP‐AQUA column (2.1 mm id × 100 mm, 2.6 µm ChromaNik Technologies Inc., Osaka, Japan) was used for chromatographic separation. All other chemicals used were of the highest available purity. The selected reaction monitoring (SRM) conditions for clozapine and its metabolites are listed in Table 2. The m/z values of precursor ion (Q1) and product ion (Q3) were set according to those in a previous study.^17^ The flow rates of the nebulizer gas, drying gas, and heating gas were 3 L/min, 10 L/min, and 10 L/min, respectively. The interface, desolvation line, and heat block temperatures were 300°C, 250°C, and 400°C, respectively, which were the default values of the LCMS‐8045 system. The mobile phase consisted of 10 mmol/L ammonium formate buffer (pH 3.6, A) and methanol (B), and an isotonic mixture of A and B (1:1, v/v) was flowed at 0.4 mL/min. The samples were processed as follows: 25 µL of sample was diluted with an equal volume of ethanol, to which 25 µL of 1 µg/mL IS solution and 75 µL acetonitrile were added. The mixtures were vortexed and centrifuged at 14,000 × *g* for 5 min at 20°C, and 50 μL of the supernatant was diluted via the addition of 75 µL of water. Finally, 1 μL of the mixture was injected into the LC–MS/MS system.

**Table 2:** Selected reaction monitoring (SRM) transitions and MS/MS operating parameters for analysis of clozapine, N‐desmethylclozapine, and clozapine N‐oxide levels.

Compound	Q1 (m/z)	Q3 (m/z)	Collision energy (eV)
Clozapine	327.0	192.1	45
N‐desmethylclozapine	313.1	192.1	46
Clozapine N‐oxide	343.1	192.1	42
Clozapine‐d_8_	335.1	192.2	48

*Note*: Q1, precursor ion; Q3, product ion.

### Genotyping of coding regions of seven pharmacokinetics‐related genes

DNA was isolated from peripheral blood samples using standard procedures. The targeted resequencing of six pharmacokinetics‐related genes (*CYP2B6*, *CYP2C19*, *CYP2D6*, *CYP3A5*, *ABCG2*, *SLCO1B1*) was performed using a MiSeq Reagent Kit v2 (Illumina, San Diego, CA, USA) with an output of 2 × 250 bp.^18^ Variants were called according to the Best Practice Workflows of GATK.^19^ The variant information was converted to the alleles of the genes registered in the Pharmacogene Variation (PhamVar) Consortium^20^ according to an in‐house method developed by Genonyx (Aichi, Japan). *CYP1A2* (rs762551) was genotyped using TaqMan probes with a QuantStudio 5 real‐time PCR system (Thermo Fisher Scientific, Waltham, MA, USA). All genotypes were in Hardy–Weinberg equilibrium.

### Statistical analysis

The clinical characteristics of the patients are presented as number or mean ± standard deviation (SD) and range. The Pearson correlation coefficient (*r*) was estimated to analyze the relationship between daily clozapine dosage or age and the plasma concentrations of clozapine, N‐desmethylclozapine, and clozapine N‐oxide. Differences between groups were tested for significance using an unpaired Student's *t*‐test. Multiple statistical comparisons were performed using Tukey's honest significant difference (HSD) test. A *p* < 0.05 was considered statistically significant. Statistics were analyzed using KaleidaGraph software (Version 4.5, Synergy Software, PA, USA).

## RESULTS

### Relationship between clozapine dose and plasma concentrations

**Figure 1:**
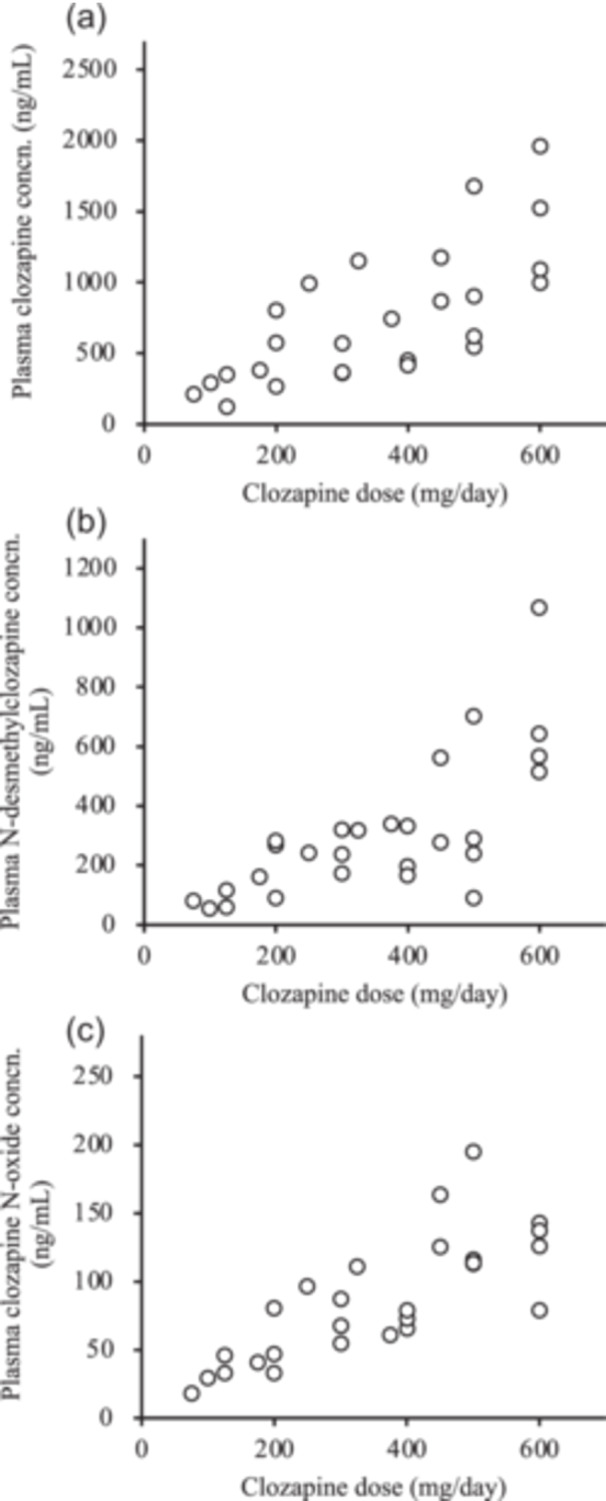
Relationship between clozapine dosage and plasma concentrations of (a) clozapine, (b) N‐desmethylclozapine, and (c) clozapine N‐oxide in 27 patients.

The clinical characteristics of the 27 patients taking clozapine are in Table 1. The plasma concentrations of clozapine, N‐desmethylclozapine, and clozapine N‐oxide widely varied. Figure 1 shows the significant positive correlation between the daily doses of clozapine and the plasma concentrations of clozapine (*r* ^2^ = 0.494, *p* < 0.001), N‐desmethylclozapine (*r* ^2^ = 0.510, *p* < 0.001), and clozapine N‐oxide (*r* ^2^ = 0.609, *p* < 0.001).

### Comparison of dose‐adjusted plasma concentrations by age and sex

The influence of age on the plasma concentrations of clozapine, N‐desmethylclozapine, and clozapine N‐oxide at a given clozapine dose was investigated. Age did not significantly correlate with the dose‐adjusted plasma concentrations of clozapine (*p* = 0.935), N‐desmethylclozapine (*p* = 0.490), or clozapine N‐oxide (*p* = 0.497).

We compared the dose‐adjusted plasma concentrations of clozapine, N‐desmethylclozapine, and clozapine N‐oxide in male and female patients; however, no significant difference was observed between the sexes (*p* = 0.596, 0.369, and 0.953 for clozapine, N‐desmethylclozapine, and clozapine N‐oxide, respectively).

### Effects of CYP enzyme genotypes

The observed genotype frequencies of *CYP1A2*, *CYP2B6*, *CYP2C19*, *CYP2D6*, and *CYP3A5* are listed in Table 1. The *CYP1A2* C‐163A polymorphism did not significantly affect the dose‐adjusted plasma concentrations of clozapine, N‐desmethylclozapine, or clozapine N‐oxide in the entire patient group (*n* = 27) (Table 3). In addition, the dose‐adjusted plasma concentrations of clozapine, N‐desmethylclozapine, and clozapine N‐oxide were not significantly different among those with other genetic polymorphisms, including *CYP2B6* (A785G, G516T), *CYP2C19* (G681A, G636A), *CYP2D6* (C100T), and *CYP3A5* (A6986G) (Table 3). The *CYP1A2* C‐163A (*1F) polymorphism is associated with a higher inducibility of CYP1A2 in smokers. Therefore, we examined the effect of smoking status on the dose‐adjusted plasma concentrations of clozapine, N‐desmethylclozapine, and clozapine N‐oxide in patients who were carriers of *CYP1A2* C‐163A (*1F) (*n* = 24). However, the dose‐adjusted plasma levels of clozapine (*p* = 0.254), N‐desmethylclozapine (*p* = 0.591), and clozapine N‐oxide (*p* = 0.677) did not significantly differ between smokers (*n* = 5) and nonsmokers (*n* = 19).

**Table 3:** Relationship between CYP polymorphism and dose‐adjusted plasma concentrations of clozapine, N‐desmethylclozapine, and clozapine N‐oxide.

		Clozapine concn./dose [(ng/mL)/mg]		N‐desmethylclozapine concn./dose [(ng/mL)/mg]		Clozapine N‐oxide concn./dose [(ng/mL)/mg]	
	*n*	Mean ± SD	*p* value	Mean ± SD	*p* value	Mean ± SD	*p* value
*CYP1A2* C‐163A			0.239		0.192		0.652
*1/*1	3	1.59 ± 0.54		0.75 ± 0.23		0.22 ± 0.05	
*1/*1F	12	2.48 ± 1.00		1.01 ± 0.46		0.25 ± 0.08	
*1F/*1F	12	1.97 ± 0.92		0.75 ± 0.24		0.26 ± 0.08	
*CYP2B6* A785G			0.321		0.210		0.583
*1/*1	13	1.96 ± 0.84		0.77 ± 0.29		0.26 ± 0.08	
*1/*4 + *4/*4	14	2.33 ± 1.04		0.95 ± 0.43		0.25 ± 0.07	
*CYP2B6* G516T			0.884		0.762		0.235
*1/*1	20	2.17 ± 0.89		0.85 ± 0.30		0.27 ± 0.07	
*1/*9 + *9/*9	7	2.10 ± 1.19		0.92 ± 0.56		0.22 ± 0.08	
*CYP2C19* G681A			0.267		0.874		0.701
*1/*1	15	1.97 ± 0.99		0.88 ± 0.43		0.25 ± 0.09	
*1/*2 + *2/*2	12	2.38 ± 0.89		0.85 ± 0.30		0.26 ± 0.07	
*CYP2C19* G636A			0.685		0.421		0.155
*1/*1	21	2.10 ± 0.90		0.90 ± 0.38		0.24 ± 0.08	
*1/*3	6	2.32 ± 1.19		0.76 ± 0.36		0.30 ± 0.07	
*CYP2D6* T733C			0.183		0.886		0.265
*1/*2	9	1.82 ± 0.87		0.87 ± 0.49		0.23 ± 0.07	
*2/*2	17	2.34 ± 0.99		0.84 ± 0.30		0.26 ± 0.08	
*CYP2D6* C100T			0.759		0.484		0.330
*1/*1	12	2.22 ± 1.12		0.92 ± 0.38		0.27 ± 0.07	
*1/*10* + 10/*10	15	2.10 ± 0.83		0.82 ± 0.37		0.24 ± 0.08	
*CYP3A5* A6986G			0.896		0.620		0.361
*1/*3	10	2.12 ± 0.90		0.82 ± 0.30		0.24 ± 0.05	
*3/*3	17	2.17 ± 1.00		0.89 ± 0.41		0.26 ± 0.09	

### Effects of ABCG2 and SLCO1B1 transporter genotypes

The genotype frequencies of *ABCG2* and *SLCO1B1* are listed in Table 1. The dose‐adjusted plasma concentrations of clozapine, N‐desmethylclozapine, and clozapine N‐oxide did not differ between the *ABCG2* 421C/C and 421C/A + A/A groups (Table 4). The *SLCO1B1* A388G polymorphism did not influence the dose‐adjusted plasma concentration of clozapine, N‐desmethylclozapine, or clozapine N‐oxide (Table 4). In addition, the dose‐adjusted plasma concentrations of clozapine and clozapine N‐oxide were not significantly affected by the *SLCO1B1* T521C polymorphism; however, carriers of the C allele of the T521C polymorphism had significantly higher dose‐adjusted concentrations of N‐desmethylclozapine than those with the wild‐type T/T genotype (Table 4).

**Table 4:** Relationship between drug transporter polymorphisms and dose‐adjusted plasma levels of clozapine, N‐desmethylclozapine, and clozapine N‐oxide.

		Clozapine concn./dose [(ng/mL)/mg]		N‐desmethylclozapine concn./dose [(ng/mL)/mg]		Clozapine N‐oxide concn./dose [(ng/mL)/mg]	
	*n*	Mean ± SD	*p* value	Mean ± SD	*p* value	Mean ± SD	*p* value
*ABCG2* C421A			0.532		0.563		0.354
C/C	10	2.02 ± 0.58		0.92 ± 0.39		0.24 ± 0.07	
C/A + A/A	17	2.23 ± 1.12		0.83 ± 0.37		0.26 ± 0.08	
*SLCO1B1* A388G			0.951		0.483		0.511
A/A	3	2.31 ± 1.49		0.65 ± 0.41		0.30 ± 0.08	
A/G	11	2.11 ± 1.04		0.85 ± 0.28		0.25 ± 0.08	
G/G	13	2.15 ± 0.83		0.94 ± 0.43		0.24 ± 0.07	
*SLCO1B1* T521C			0.775		0.033		0.692
T/T	22	2.13 ± 0.99		0.81 ± 0.38		0.25 ± 0.08	
T/C	5	2.26 ± 0.83		1.11 ± 0.21		0.27 ± 0.09	

### Multiple regression analysis

A multiple regression analysis including sex, age, smoking status, and genotypes was conducted for the dose‐adjusted plasma concentrations of clozapine, N‐desmethylclozapine, and clozapine N‐oxide. Only the SLCO1B1 T521C genotype remained significant, which was consistent with the previous results.

## DISCUSSION

We examined the effects of clozapine dose as well as patient age, sex, and genetic polymorphisms of CYP isoforms and drug transporters on the plasma concentrations of clozapine, N‐desmethylclozapine, and clozapine N‐oxide in 27 Japanese patients with schizophrenia. The plasma concentrations significantly positively correlated with the daily clozapine dose, aligning with the findings of previous studies that the daily clozapine dose positively correlated with the plasma concentrations of clozapine and/or N‐desmethylclozapine in patients taking clozapine.^21^, ^22^, ^23^, ^24^ Yada et al.^25^ demonstrated that the daily clozapine dosage was positively related to the plasma concentrations of clozapine and N‐desmethylclozapine in Japanese patients with treatment‐resistant schizophrenia. Our observations were consistent with the positive linear relationship between the dose and plasma concentrations of clozapine and N‐desmethylclozapine. In addition, we measured another clozapine metabolite, clozapine N‐oxide. Similar to clozapine and N‐desmethylclozapine, a significant positive correlation was observed between the daily clozapine dose and plasma concentration of clozapine N‐oxide. To the best of our knowledge, this is the first report of a significant positive correlation between the daily clozapine dose and plasma concentrations of clozapine N‐oxide in Japanese patients.

Age and the plasma concentrations of clozapine and N‐desmethylclozapine were not significantly correlated. This observation is consistent with those of studies reporting no significant age‐related differences in plasma clozapine and/or N‐desmethylclozapine levels.^22^, ^23^, ^26^, ^27^, ^28^ Other studies found that age affects the plasma concentration of clozapine^21^, ^29^, ^30^; however, the influence of age on the plasma concentration of clozapine N‐oxide was not examined. Age and the plasma concentrations of clozapine N‐oxide, clozapine, or N‐desmethylclozapine were not significantly correlated in the patients in this study.

Significant relationships between the plasma clozapine level and sex have been reported.^21^, ^22^, ^27^, ^29^, ^30^ However, no statistical significance in the plasma levels of clozapine and N‐desmethylclozapine was found between men and women in other studies.^23^, ^24^, ^26^, ^28^ Lane et al.^29^ found no significant sex‐related differences in clozapine N‐oxide levels, which is consistent with our finding.

The timing of blood sampling and medication may have influenced the plasma concentration results. To address this, we divided the participants into two groups: the “same‐day” group (within 6 h of the last dose, *n* = 6) and the “previous‐day” group (within 6–18 h of the last dose, *n* = 21). A Welch two‐sample *t*‐test was performed, and no significant differences in the plasma concentrations of the three substances were observed between the two groups (*p* > 0.05, respectively). While strict control of the timing of medication and blood sampling should ideally be ensured, the inability to achieve this represents a major limitation of this study. Although the results of the *t*‐test may have been influenced by the limited sample size, the lack of significant differences between the two groups suggests that the interpretation of the plasma concentration data does not require substantial modification.

We found no significant differences in the plasma concentrations of clozapine and its metabolites, *CYP1A2*, *CYP2B6*, *CYP2C19*, *CYP2D6*, and *CYP3A5*, among the patients. Akamine et al.^31^ found that *CYP2D6* and *CYP3A5* polymorphisms were not associated with plasma levels of clozapine and N‐desmethylclozapine in Japanese patients. Tóth et al.^32^ reported that the *CYP2C19* or *CYP2D6* genotypes did not affect the serum concentration of clozapine. In contrast, Jaquenoud et al.^33^ found that the *CYP2C19* genotype strongly influenced the plasma concentration of clozapine but not that of N‐desmethylclozapine, whereas the *CYP2B6*, *CYP2C9*, *CYP2D6*, *CYP3A5*, and *CYP3A7* genotypes did not affect the plasma levels of clozapine or N‐desmethylclozapine. Kootstra‐Ros et al.^34^ found no significant correlation between the *CYP1A2* genotypes (*1F, *1C, *1D) and clozapine clearance. In contrast, Olsson et al.^28^ reported a significant association between the AA genotype of *CYP1A2* C‐163A (*1F) and low plasma clozapine concentrations. Jaquenoud et al.^33^ found a significant correlation between CYP1A2 activity and the plasma concentration of clozapine; however, the CYP1A2*1F polymorphism did not affect the plasma clozapine concentrations in the patients who were smokers. We also observed no significant differences in the dose‐adjusted plasma levels of clozapine, N‐desmethylclozapine, and clozapine N‐oxide between smokers and nonsmokers among the *CYP1A2* C‐163A (*1F) carriers.

We observed no significant differences in the plasma concentrations of clozapine, N‐desmethylclozapine, and clozapine N‐oxide between *ABCG2* 421C/C and 421C/A + A/A. In contrast, Akamine et al.^31^ found that plasma clozapine levels were significantly higher in patients with the *ABCG2* 421A allele than in those with the *ABCG2* 421C/C genotype, indicating that the breast cancer resistance protein (BCRP/ABCG2) affects clozapine exposure. The reason for the inconsistency of the effects of the *ABCG2* genotype among studies is currently unclear. Akamine et al.^31^ found no significant differences in the plasma levels of N‐desmethylclozapine between the *ABCG2* 421C/A + A/A and *ABCG2* 421C/C genotypes, which is consistent with our observations.

The levels of N‐desmethylclozapine were significantly higher in patients who were carriers of the C allele of *SLCO1B1* T521C than in those with the wild‐type T/T genotype; however, the plasma levels of clozapine and clozapine N‐oxide were not considerably affected by the *SLCO1B1* T521C polymorphism. Additionally, the *SLCO1B1* A388G polymorphism did not significantly affect the plasma concentrations of clozapine, N‐desmethylclozapine, or clozapine N‐oxide. In the present sample, all five individuals with *5 (T521C) were found to carry *37 (A388G), indicating that the individuals with *5 were equal to those with *15 (A388G and T521C together). A genome‐wide association study^8^ identified an association between the genetic variant rs149104283 and the risk of clozapine‐induced agranulocytosis/granulocytopenia. The rs149104283 SNP is located within a genomic region on chromosome 12, encoding *SLCO1B3*, *SLCO1B7*, and *SLCO1B1*. Therefore, the genetic variant *SLCO1B3* and/or *SLCO1B1* may enhance the induction of clozapine‐associated neutropenia via a pharmacokinetic mechanism because the liver‐specific organic anion transporter polypeptides (OATPs), SLCO1B1 and SLCO1B3, mediate the basolateral uptake of organic anions in hepatocytes. Saito et al.^35^ found a modest association between the risk of clozapine‐associated neutropenia and rs11045434 in the genomic region of *SLCO1C1* and upstream of *SLCO1B3*. These findings indicate the involvement of liver‐specific OATPs that facilitate uptake from the portal vein into hepatocytes in clozapine‐associated neutropenia. However, Dickens et al.^36^ reported that clozapine was neither a substrate nor an inhibitor of SLCO1B1 or SLCO1B3, suggesting that the hepatic uptake of clozapine is not mediated by these transporters. Sato et al.^37^ found that patients with *SLCO* gene variants are likely to be highly exposed to clozapine and/or N‐desmethylclozapine and that clozapine was a substrate of SLCO1B1, indicating that clozapine is an SLCO1B1 substrate and the presence of SNPs in OATPs alters the pharmacokinetics of clozapine. Park et al.^38^ reported that radioactive ^11^C‐clozapine accumulated the most in the liver, indicating the involvement of a specific transport mechanism in the hepatic accumulation of clozapine and/or its metabolites, N‐desmethylclozapine and clozapine N‐oxide.

Henning et al.^39^ found that clozapine uptake in HL‐60 human promyelocytic leukemia cells was saturable as well as energy‐ and temperature‐dependent, suggesting that clozapine membrane passage occurs via a carrier mechanism. Bergemann et al.^40^ reported that patients who developed leukocytopenia had clozapine concentrations in the leukocytes that were approximately eight times higher than in patients receiving clozapine, indicating the presence of a clozapine transporter in leukocytes. Alterations or dysfunctions in the clozapine‐specific transporter system within the cell membrane may contribute to the onset of clozapine‐induced leukocytopenia and/or agranulocytosis.

Veys et al.^41^ reported that N‐desmethylclozapine more strongly affected mitotic myeloid compartment (maturation to the metamyelocyte) than clozapine and clozapine N‐oxide and was therefore the more likely cause of agranulocytosis. Gerson et al.^42^ reported that N‐desmethylclozapine had 4‐ to 10‐fold greater toxicity toward hematopoietic progenitors in the bone marrow compared to clozapine and its metabolites, indicating that N‐desmethylclozapine is harmful to marrow precursors. The potential target of N‐desmethylclozapine appears to be an early hematopoietic precursor rather than a committed granulocyte stem cell. This aligns with findings that N‐desmethylclozapine was more cytotoxic to cells within the mitotic myeloid compartment than to more differentiated myeloid cells.^41^ Thus, N‐desmethylclozapine may pose greater toxicity to the bone marrow than the parent compound or its other metabolites.

## CONCLUSIONS

The relationships between age, sex, genetic polymorphisms in pharmacokinetics‐related factors, and the plasma concentrations of clozapine and its metabolites (N‐desmethylclozapine and clozapine N‐oxide) were studied in Japanese patients with schizophrenia. We found that the *SLCO1B1* T521C polymorphism strongly impacted the pharmacokinetics of N‐desmethylclozapine. We do not believe that the significant associations observed or the lack of associations in this small sample size have yielded clinically applicable results. However, we hope that in the future, accumulating similar studies with limited sample sizes and conducting integrative research designs, such as meta‐analyses, will lead to cases where drug dosages can be predicted in advance. Our findings indicate that tailoring medication depending on the *SLCO1B1* genotype and plasma N‐desmethylclozapine levels may be useful for improving the outcomes of individual clozapine therapy.

## AUTHOR CONTRIBUTIONS

Shinya Kinoshita, Hideyuki Motohashi, Keiichiro Nishida, Tetsufumi Kanazawa, and Junya Nagai wrote the manuscript. Tetsufumi Kanazawa and Junya Nagai conceptualized and designed the research. Shinya Kinoshita, Hideyuki Motohashi, Keiichiro Nishida, and Seiichiro Tarutani performed the research and analyzed the data. All authors contributed to the revision of the manuscript and gave final approval of the version to be published.

## CONFLICT OF INTEREST STATEMENT

The authors declare no conflicts of interest.

## ETHICS APPROVAL STATEMENT

This study was conducted in accordance with the Declaration of Helsinki and approved by the Ethics Committee of Osaka Medical and Pharmaceutical University (approval No. 2021‐147‐2).

## PATIENT CONSENT STATEMENT

All patients provided written informed consent to participate in this study.

## CLINICAL TRIAL REGISTRATION

N/A.

## Data Availability

The data that support the findings of this study are available on request from the corresponding author. The data are not publicly available due to privacy or ethical restrictions.
